# TERTIAN: Clinical Endpoint Prediction in ICU via Time-Aware Transformer-Based Hierarchical Attention Network

**DOI:** 10.1155/2022/4207940

**Published:** 2022-12-16

**Authors:** Ying An, Yang Liu, Xianlai Chen, Yu Sheng

**Affiliations:** ^1^Big Data Institute, Central South University, Changsha, Hunan, China; ^2^School of Computer Science and Engineering, Central South University, Changsha, Hunan, China

## Abstract

Accurately predicting the clinical endpoint in ICU based on the patient's electronic medical records (EMRs) is essential for the timely treatment of critically ill patients and allocation of medical resources. However, the patient's EMRs usually consist of a large amount of heterogeneous multivariate time series data such as laboratory tests and vital signs, which are produced irregularly. Most existing methods fail to effectively model the time irregularity inherent in longitudinal patient medical records and capture the interrelationships among different types of data. To tackle these limitations, we propose a novel time-aware transformer-based hierarchical attention network (TERTIAN) for clinical endpoint prediction. In this model, a time-aware transformer is introduced to learn the personalized irregular temporal patterns of medical events, and a hierarchical attention mechanism is deployed to get the accurate patient fusion representation by comprehensively mining the interactions and correlations among multiple types of medical data. We evaluate our model on the MIMIC-III dataset and MIMIC-IV dataset for the task of mortality prediction, and the results show that TERTIAN achieves higher performance than state-of-the-art approaches.

## 1. Introduction

Intensive care unit (ICU) aims to provide comprehensive and reliable treatments for critically ill patients. It gathers the most important resources of the hospital including medical equipment and staff. Since the first ICU was established in the United States in the 1960s, the amount of ICU has maintained a rapid growth trend and been popularized all over the world [1]. In 2019, the number of patients admitted to ICU in China reached 2.11 million, and the in-hospital mortality rate for critically ill patients was 8.3%. Worldwide, the mortality rate of ICU patients remains high, ranging from 10% to 20%, and this number is extremely susceptible to the scarcity of the hospital resources and the health status of the patients [2]. Patient's endpoint prediction in the ICU is closely related to intervention options, nursing plan formulation, and resource allocation. Accurate evaluation of patient mortality risk and early identification of patients with poor prognosis can help doctors assess the patient's condition, which is the key to improve the survival rate and physiological outcome of the patient. In order to adopt more efficient and cost-effective manners for diagnosis and treatment, ICU mortality prediction is helpful for doctors to assess the patient's condition. However, it is difficult to estimate the patient's risk of death based on the subjective experience of the clinician alone [2].

The rapid development of hospital informatization has promoted the digitization of medical records. A large amount of electronic medical records (EMRs) are available for medical research and applications. In the past decades, a large number of scholars have used EMRs data to carry out a series of studies on personal health evaluation and clinical prediction [3, 4]. Many scoring models based on statistical machine learning [2, 5–10] have been proposed and become the criteria for severity and mortality risk prediction, such as Acute Physiology and Chronic Health Evaluation (APACHE) [5] and Simplified Acute Physiology Score (SAPS) [7]. However, most of these models adopted traditional human-intervention feature engineering which is highly dependent on the knowledge and experience of researchers. Moreover, they are often limited to the modeling of linear decision boundaries and lack the ability to capture complex nonlinear relationships and temporal information. Hence, the scoring models are unable to yield satisfactory prediction performance in practical applications.

In recent years, deep learning has been widely used in electronic medical record mining and shown promising performance. However, there are also many challenging issues that need to be resolved urgently. One of the issues is irregular time series modeling in EMRs. In the medical field, different clinical events are usually occurred or recorded at different frequencies. In addition, the irregular occurrence of medical events is usually related to the patient's health status. Therefore, how to effectively mine the unique progression patterns through time for different patients from the multivariate irregular time series data contained in EMRs is particularly important. However, most existing approaches often ignore the irregularity of the time interval between medical events [11–17], or simply assume that the more recent medical events weight more than the previous ones and adjust the impact of time interval to medical prediction by using a time-related decay function [18–21]. It is undeniable that the frequency of medical visits can reflect the health status of patients to a certain extent, and medical events that occur at different times may also have different effects on the development of diseases. However, the influence weights of different types of historical medical events are not always decayed over time but may have completely different changing patterns. The attention mechanism can dynamically control the memory decay according to the calculated attention score, thus generating an adaptive decay mode that is consistent with the characteristics of disease development [22]. Therefore, we adopt a time-aware transformer to learn the pattern of each clinical event over time. In the transformer structure, the multihead self-attention mechanism is used to enhance the ability of modeling irregular time series. Compared with the exponential decay rate of the standard LSTM, it brings much slower memory decays, which is more conductive to the capture of long-term dependencies in time series data.

Another challenging issue is heterogeneous data fusion representation. EMRs contain a wealth of heterogeneous data related to patient conditions, including demographic statistics, diagnosis, laboratory test results, prescriptions, and clinical notes. These heterogeneous data are interrelated with each other and are reflection of the patient's health status from different perspectives. Therefore, the key of heterogeneous data fusion representation is to obtain the respective characteristics of various data while capturing the potential correlation between them. In the existing methods, heterogeneous data fusion is often implemented in two main ways. One is data-level fusion, which directly fuses various types of raw data in the input layer of the model [18, 23]. The other is representation-level fusion, which fuses the feature representations of different types of data by performing concatenation or element wise operation (summation, average, and multiplying) [24–29]. Although these methods have achieved certain performance improvements, they are not capable of achieving effective heterogeneous data fusion representation to capture the differences and correlations among various data at the same time.

To address the issues mentioned above, we propose a time-aware transformer-based hierarchical attention network (TERTIAN) to tackle the mortality prediction problem inside ICU. There are two key features in our method. One is to model irregular time series data to learn the irregular temporal pattern of each sample, which helps to more accurately express the patient's disease progression. Another is using a hierarchical attention mechanism to fuse different types of clinical data layer by layer according to the interactions between the patient's clinical examination (e.g., laboratory test and vital signs) and treatment (e.g., prescription). This layer-by-layer fusion approach can make use of the potential interaction between different types of data to comprehensively represent the patient's health status from multiple perspectives. Our main contributions are summarized as follows:

- We design an irregular temporal pattern learning method, which uses the time-aware transformer to learn the pattern of each clinical event over time. Such a temporal pattern preserves the specificity of each clinical event and each patient.
- We propose a hierarchical attention mechanism to fuse different types of data. In the first layer, the representation of prescription is used as a key vector and the representation of each clinical examination is used as query vector to capture the interaction between them. Then, the second-layer attention mechanism is utilized to integrate different types of clinical data and obtain the final patient fusion representation for prediction.
- We conduct the mortality prediction task on two real-world datasets (MIMIC-III dataset and MIMIC-IV dataset) to verify the performance of our method. Ablation studies and model analysis validate the effectiveness of the proposed model.

The rest of this paper is organized as follows. We introduce deep learning methods applied to clinical medical prediction in Section 2; we depict the methodology of TERTIAN in Section 3; we present the details of experimental implementation and discuss the experiments results in Section 4; finally, the conclusions are given in Section 5.

## 2. Related Works

In clinical practice, the assessment of ICU patients is usually based on APACHE, SAPS, and other scoring systems. However, these scoring systems are weak in generalization. They usually consider the patient's current vital signs and reaction test results, while ignoring the temporality of the patient's medical records. In recent years, due to the popularity of EMRs containing multiple heterogeneous time series data and the obvious advantages of deep learning methods in many fields, a large number of scholars have used EMR data to carry out a series of clinical prediction studies based on deep learning models. In order to solve the problem of time series modeling of EMR data, recurrent neural network (RNN) and its variants (LSTM [30] and GRU [31]), temporal convolutional network (TCN) [32], and other deep neural network models have achieved promising performance in various applications. For example, Choi et al. [16] proposed a multilabel prediction model based on recurrent neural networks, which uses the longitudinal time stamped EHR data (e.g., diagnosis codes, medication codes, or procedure codes) to predict the diagnosis and medication categories in the subsequent visit. Nguyen et al. [11] presented an end-to-end learning system that can automatically extract features from medical records and predict patient's risk of illness in the future. In this model, a medical record is converted into a sequence of discrete elements, and a convolutional neural network is utilized to discover the predictive local clinical motifs to stratify the risk. Although these methods can capture the temporal information in EMR data to a certain extent, most of them fail to fully consider the irregularity of the time interval between different medical events.

Recently, many modifications to the traditional RNN/CNN model have been proposed to realize the irregular time series modeling for EMR data. Suo et al. [33] built a novel time fusion CNN framework for personalized disease prediction, which can capture not only the local temporal relationships but also the contributions from each time interval. Baytas et al. [21] proposed a novel LSTM unit called time-aware LSTM (T-LSTM) to tackle time irregularities in longitudinal patient records. In this model, the elapsed time is transformed into a weight by using a time decay function, and then the sequential records of patients are mapped to a representation capturing the dependencies between the elements in the presence of time irregularities. Similarly, Bai et al. [19] presented an interpretable deep learning model called TimeLine for diagnosis prediction of future admissions. In TimeLine, a time-aware disease progression function which depends on the specific medical code and the elapsed time between visits is applied to model how much each recorded disease influences the subsequent visits. In addition, Yang et al. [34] also proposed a multiseries time-aware TICC for disease progression modeling, which incorporates multiseries nature and irregular time interval of EHRs. They incorporated time-awareness for the consistency between consecutive series, that is, introducing a nonlinear monotonic decreasing function to solve the problem of TICC ignores the intervals and encourages the consecutive records to be assigned into the same cluster. Most of the above-mentioned approaches handle time irregularity in patient's medical record sequences by means of information decay, which gives recent medical events more weights and reduces the weight of previous medical events according to the time elapsed. However, these methods cannot accurately model the patient's condition progression pattern since the influence of medical events does not necessarily change monotonically over time.

Additionally, considering that the development of patients' condition is a complex process closely related to multiple factors, the deep learning methods based on EMRs have gradually evolved from the single-view learning for a single-type of data to the multiview learning for heterogeneous data. In recent years, researchers have made a lot of attempts on how to obtain a comprehensive patient representation by effectively fusing various clinical data (laboratory tests, diagnosis, medication records, physical examinations, etc.) in EMRs. In the existing studies, the most common data fusion method is data-level fusion directly concatenating heterogeneous raw data in model's input layer. Che et al. [18] proposed a deep learning model based on gated recurrent unit (GRU), which combines 99 time series features (e.g., input events, output events, lab events, and prescription events) to predict the possibility of the patient death in the hospital. Liu et al. [23] presented a model for learning the joint representation of heterogeneous temporal events to predict clinical endpoints. In this model, each heterogeneous medical event is defined as a triple one consisting of the event category, event attribute value, and event timestamp. Then, the heterogeneous event sequence of patient is fed to a novel RNN model called HE-LSTM to learn the representation.

Another data fusion method is representation-level fusion, which usually learns the representation of different types of data separately first and combines the feature representations through concatenation, element-wise operation, or other neural networks. For example, Ding et al. [35] proposed a UGI cancer screening approach based on semantic-level dual-modality data fusion. In this modal, the features of medical images are extracted by customized CNNs, and the textual records features are extracted by word2vec and self-attention. Then, the medical image features and textual features are concatenated and the fused information is used to obtain the weights of each feature channel in CNNs. Finally, the multimodal fusion representation is obtained by the cascading operation of the weighted medical image features and the textual features. Zhang et al. [28] proposed a Multilayer Multiview Classification (ML-MVC) approach for Alzheimer's disease diagnosis, which introduces a middle layer model for feature extraction with the kernel technique to account for nonlinearity and jointly stacks kernel matrices to capture the complementary information from multiple views. Qiao et al. [27] proposed Multimodal Attentional Neural Networks (MNN) to model the medical codes and clinical notes in a unified framework. It applies a CNN and a bidirectional GRU network to separately learn the textural feature representation and medical code feature representation from different types of inputs. Then, the final multimodal feature representation is generated by a deep feature mixture module and fed into an attentional bidirectional RNN to model sequential clinical visits. Similarly, Ma et al. [20] proposed a health status representation framework called ConCare, which jointly considers static baseline information, sequential dynamic features, and the impact of the time interval as personal health context for mortality prediction. It learns the representation of different feature sequences via separate GRUs and adaptively captures the effect of time intervals between records of each feature by time-aware attention. Then, a feature encoder based on multihead self-attention is introduced to combine different clinical information. Although these approaches are proved to have some improvements in performance, most of them are not able to fully capture the interactions and interrelationships among various clinical data. Therefore, the deep fusion of heterogeneous features still cannot be achieved effectively.

## 3. TERTIAN

In this section, we first introduce the definition of the problem and some basic notations used in this paper. Then, we describe the proposed model in detail.

### 3.1. Problem Formulation

Assume that *P*={*p*_1,_*p*_2,_ ⋯ , *p*_|*P*|_} represents the set of patients, where |*P*| is the total number of patients. The patient's clinical records used in this paper consist of three types of data: prescription information *M*, laboratory test results *X*^*l*^, and vital signs *X*^*r*^. As a result, the clinical records of each patient *p*_*i*_ can be denoted as (*M*, *X*^*l*^, *X*^*r*^). For each patient, the prescription information *M* consists of a set of prescription codes *M*={*m*_1_, *m*_2_, ⋯, *m*_*Z*_}, where each element *m*_*i*_ represents a prescription code and *Z* is the total number of prescription codes appearing in the patient's clinical records. Both laboratory test results *X*^*l*^ and vital signs *X*^*r*^ are dynamic monitoring information, which contains multiple related clinical variables, and each variable can be expressed as a time-ordered sequence composed of a set of continuous recorded values. We denote *X*^*C*^ as the dynamic monitoring information, where *C* ∈ (*l*, *r*) is the category label, namely, *C*=*l* represents laboratory test results *X*^*l*^ and *C*=*r* represents vital signs *X*^*r*^. Then, any dynamic monitoring information (laboratory test results or vital signs) can be uniformly expressed as*X*^*C*^=[*x*_1_^*C*^, *x*_2_^*C*^, ⋯, *x*_|*X*^*C*^|_^*C*^], where |*X*^*C*^| is the number of clinical variables contained in *X*^*C*^. Let *x*_*i*_^*C*^=(*x*_*i*1_^*C*^, *x*_*i*2_^*C*^, ⋯, *x*_*iT*_^*C*^) represent the sequence corresponding to any dynamic monitoring variable *x*_*i*_^*C*^, where *x*_*it*_^*C*^ is the *t*th observed value and *T* is the length of sequence. We denote *d*_*it*_^*C*^ as the timestamp of the *t*th observation *x*_*it*_^*C*^, and *δ*_*it*_^*C*^=*d*_*it*_^*C*^ − *d*_*i*(*t* − 1)_^*C*^ represents the interval between any two adjacent records, where *δ*_*i*1_^*C*^=0. Since different variables may be recorded at irregular times, for any variable *x*_*i*_^*C*^, we set *x*_*it*_^*C*^=0 and *d*_*it*_^*C*^=*d*_*i*(*t* − 1)_^*C*^ when its value is missing at the *t*th observation.

The purpose of our study is to predict the ICU mortality by using prescription information *M*, laboratory tests *X*^*l*^, and vital signs *X*^*r*^ for the patient's first 48 hours since admission. In the absence of ambiguity, we omit the category label *C* of the dynamic monitoring information to simplify the representation in the rest of this paper. In addition, definitions and descriptions of common symbols in TERTIAN are presented in Table 1. The details of TERTIAN are presented in the following section.

### 3.2. Model Overview

**Figure 1:**
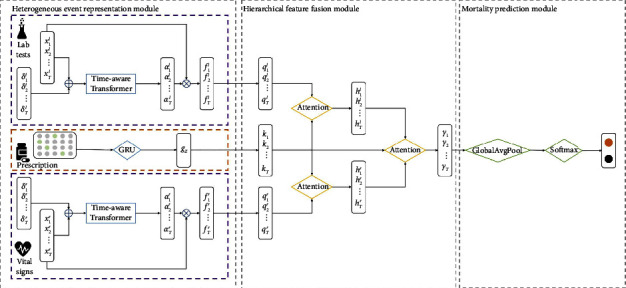
The architecture of TERTIAN.

Figure 1 shows a general framework of our proposed model TERTIAN. It consists of three main components: heterogeneous event representation module, hierarchical feature fusion module, and mortality prediction module. In the heterogeneous event representation module, we separately apply multiple deep representation learning models to capture the unique temporal patterns from different types of data such as laboratory test results, vital signs, and prescriptions. Then, the feature representations of the data are fed into the hierarchical feature fusion module, which uses a two-layer attention mechanism to mine their interactions and correlations and obtain the final patient fusion representation. Finally, the mortality prediction module is applied to obtain the final prediction results.

### 3.3. Heterogeneous Event Representation Module

To preserve the specificity of clinical information hidden in different types of data, in this module, we separately apply two deep learning models to learn the unique temporal patterns of various data sequences. For laboratory tests and vital signs, considering that the irregularity of time interval usually contains a lot of important information related to the development of patient's health status, we use a time-aware transformer to automatically learn personalized irregular temporal pattern from time series. Inspired by HiTANet [36], we embed time information into time series data by element-wise addition. In order to fully preserve the difference between the sampling time of the feature data, we adopt the feature-level time interval representation which maintains the time interval for each feature since its last observation. Specifically, we first make the time interval *δ* and the dynamic monitoring time series *X* in the same latent space by normalizing the time information. The normalized time interval vector *s*_*t*_ is obtained via equation (1):

(1) $$\begin{array}{c} s_{t}=W_{s}\left(1-\tan\;h\left({\left(W_{\delta }\delta_{t}+b_{\delta }\right)}^{2}\right)\right)+b_{s}\text{,} \end{array}$$

where *W*_*δ*_ ∈ *ℝ*^*a*^, *b*_*δ*_ ∈ *ℝ*^*a*^, *W*_*s*_ ∈ *ℝ*^*a*×*a*^, and *b*_*s*_ ∈ *ℝ*^*a*^ are all parameters. *δ*_*t*_ is a time interval vector that represents the time interval between two adjacent nonmissing values of each feature, *a* is the dimensionality of *s*_*t*_ which also represents the number of features in our work.

Then, any dynamic monitoring variable vector *x*_*t*_ and its corresponding time interval embedding vector *s*_*t*_ will be calculated via equation (2) to obtain the input vector *e*_*t*_. Thus, the dynamic monitoring information *X* is mapped into the input vector sequence E=[*e*_1_, *e*_2_, ⋯, *e*_*T*_]:

(2) $$\begin{array}{c} e_{t}=x_{t}+s_{t}\text{.} \end{array}$$

Next, we feed the input matrix *E*=[*e*_1_, *e*_2_, ⋯, *e*_*T*_] to the transformer. The transformer is a deep learning architecture based on attention mechanisms and consists of an encoder and a decoder. The core components of the encoder and decoder are multihead self-attention and feed-forward network. In the encoder, the input matrix *E* passes through the multihead self-attention layer and the feed-forward layer with addition and normalization operation. The output of the encoder will be used as the input of the decoder, and the execution process of the decoder is similar to that of the encoder. Through this encoder-decoder structure of the transformer to get the hidden representation, *φ*=[*φ*_1_, *φ*_2_, ⋯, *φ*_*T*_] and then derive the time-aware attention weights *α*=[*α*_1_, *α*_2_, ⋯, *α*_*T*_] via equations (3) and (4). Then, based on the weights, the time-aware contextual feature representation *f*_*t*_ is obtained by equation (5):

(3) $$\begin{array}{c} \varphi =Transformer\left(e_{1},e_{2},\cdots ,e_{T}\right)\text{,} \end{array}$$

(4) $$\begin{array}{c} \alpha =sigmoid\left(\varphi \right)\text{,} \end{array}$$

(5) $$\begin{array}{c} f_{t}=\alpha_{t}*x_{t}\text{.} \end{array}$$

For prescription information *M*={*m*_1_, *m*_2_, ⋯, *m*_*Z*_}, the time information of each prescription code is not recorded in detail. Therefore, we simply treat prescription information as a time-ordered code sequence without considering their time interval information. The GRU is an improved version of RNN which addresses the problem of vanishing gradient and achieves good performance in sequential form. In this paper, we utilize a GRU module with two unidirectional GRU layers. The GRU module takes *M* as input to extract the temporal pattern hidden in prescription information and obtains the corresponding temporal feature representation [*g*_1_, *g*_2_, ⋯, *g*_*Z*_]. Specifically, firstly, at the *z*th time-step, the GRU units can decide how to combine the previous hidden state *g*_*z*−1_ and the current input *m*_*z*_ by using the reset mechanism with equation (6). At the same time, the update gate up *d*_*z*_ controls how much of the previous memory content is to be forgotten and how much of the new memory content is to be added with equation (7). Then, the new candidate memory content ${\tilde{g}}_{z}$ is computed considering the reset gate res_*z*_ with equation (8). Finally, the new memory state *g*_*z*_ is obtained through the update mechanism as equation (9):

(6) $$\begin{array}{c} {res}_{z}=sigmoid\left(W_{res}\left[g_{z-1},m_{z}\right]\right)\text{,} \end{array}$$

(7) $$\begin{array}{c} {upd}_{z}=sigmoid\left(W_{upd}\left[g_{z-1},m_{z}\right]\right)\text{,} \end{array}$$

(8) $$\begin{array}{c} {\tilde{g}}_{t}=\tan\;h\left(W_{\tilde{g}}\left[{{res}_{z}*g}_{z-1},m_{z}\right]\right)\text{,} \end{array}$$

(9) $$\begin{array}{c} g_{t}=\left(1-{upd}_{z}\right)*g_{z-1}+{upd}_{z}*{\tilde{g}}_{z}\text{,} \end{array}$$

where matrices *W*_res_, *W*_up*d*_, and $W_{\tilde{g}}$ are model parameters.

### 3.4. Hierarchical Feature Fusion Module

Through the heterogeneous event representation module, we obtain the corresponding contextual representations *F*^*l*^=[*f*_1_^*l*^, *f*_2_^*l*^, ⋯, *f*_*T*_^*l*^], *F*^*r*^=[*f*_1_^*r*^, *f*_2_^*r*^, ⋯, *f*_*T*_^*r*^], and *g*_*Z*_ from three types of data (laboratory test results, vital signs, and prescription information). Then, we design a two-layer attention mechanism to capture the interdependencies among different types of data and obtain the final fusion representation.

Considering that in the clinical process, doctors usually prescribe or adjust drug prescriptions based on the patient's dynamic monitoring results, such as laboratory tests and vital signs. At the same time, the effects of the prescribed drugs are reflected in the patient's subsequent dynamic monitoring results. Therefore, we use a two-layer attention mechanism to discover the interrelationships between different clinical information. Specifically, in the first layer, the laboratory tests representation *f*_*t*_^*l*^ is projected into the query vector *q*_*t*_^*l*^, and the prescription information representation *g*_*Z*_ is projected into the key vector *k*_*t*_. Then, we calculate the attention weights *β*_*t*_^*l*^*t* to capture the correlation between prescription information and laboratory test results. The calculation process is described as follows:

(10) $$\begin{array}{c} q_{t}^{l}=W_{q^{l}}f_{t}+b_{q^{l}}\text{,} \end{array}$$

(11) $$\begin{array}{c} k_{t}=W_{k}g_{Z}+b_{k}\text{,} \end{array}$$

(12) $$\begin{array}{c} \beta_{t}^{l}=softmax\left(\left[\frac{q_{t}^{l}k_{1}}{\sqrt{d}},\frac{q_{t}^{l}k_{2}}{\sqrt{d}},\cdots ,\frac{q_{t}^{l}k_{T}}{\sqrt{d}}\right]\right)\text{,} \end{array}$$

where *W*_*q*^*l*^_ ∈ *ℝ*^*q*×*l*^ and *W*_*k*_ ∈ *ℝ*^*q*×*m*^ are the projection matrices and *b*_*ql*_ ∈ *ℝ*^*q*^ and *b*_*k*_ ∈ *R*^*q*^ are the bias vectors.

Thus, the mixed vector *h*_*t*_^*l*^ that integrates the relationship between the prescription information and the laboratory test results can be obtained via equation (13), and the sequence representation becomes *H*^*l*^=[*h*_1_^*l*^, *h*_2_^*l*^, ⋯, *h*_*T*_^*l*^]. Using the same method, we get the mixed vector representation sequence *H*^*r*^ of prescription information and vital signs:

(13) $$\begin{array}{c} h_{t}^{l}=Attention\left(q_{t}^{l},k_{t},f_{t}^{l}\right)=\overset{T}{\underset{j=1}{\sum }}\beta_{t}^{l}f_{j}^{l}\text{.} \end{array}$$

In the second layer, we further merge two mixed vector representations (*H*^*l*^ and *H*^*r*^) obtained in the first layer with the key vectors *K* to get the final patient representation, where *K* is composed of the vector *k*_*t*_ by equation (11) repeated *T* times. Here, *H*^*r*^, *K*, and *H*^*l*^ are used to generate query vectors, key vectors, and value vectors, respectively, and fed into the attention function to obtain the final patient fusion representation Γ. The calculation process is as follows:

(14) $$\begin{array}{c} \Gamma =\left[\gamma_{1},\gamma_{1},\cdots ,\gamma_{T}\right]=Attention\left(H^{r},K,H^{l}\right)\text{.} \end{array}$$

### 3.5. Mortality Prediction Module

The final fusion representation Γ is projected into a vector *γ*′*ϵℝ*^256^ by global average pooling. Finally, a simple linear layer with the softmax activation function is used to make a binary prediction as follows:

(15) $$\begin{array}{c} y^{\prime }=softmax\left(W_{y}\gamma^{\prime }+b_{y}\right)\text{,} \end{array}$$

where *W*_*y*_ ∈ *ℝ*^*u*×*q*^ and *b*_*y*_ ∈ *ℝ*^*u*^ are trainable parameters, respectively representing the weight and bias. *u* is the number of categories, and *u*=2 in this paper.

Here, the cross-entropy is used to calculate the loss between the true value *y* and the prediction label *y*′.

(16) $$\begin{array}{c} L=-\frac{1}{\left|P\right|}\overset{\left|P\right|}{\underset{i=1}{\sum }}\left[y_{i}\ln y_{i}^{\prime }+\left(1-y_{i}\right)\ln\;\left(1-y_{i}^{\prime }\right)\right]\text{,} \end{array}$$

where |*P*| is the total number of patients.

## 4. Experiments

### 4.1. Dataset Description

We conduct all the experiments on the Medical Information Mart for Intensive Care III (MIMIC-III) [37] dataset and the Medical Information Mart for Intensive Care IV (MIMIC-IV) [38] dataset. MIMIC-III is a large and freely available database comprising deidentified health-related data associated with more than forty thousand patients who stayed in critical care units of the Beth Israel Deaconess Medical Center between 2001 and 2012. MIMIC-IV also records the comprehensive information of patients in a medical center in the United States and has made many improvements and expansions on the basis of MIMIC-III. It not only records the data of the intensive care unit but also includes the information of emergency and general hospitalization. The MIMIC-IV dataset recorded a total of 256,878 patients' visit information, including more than 50,000 patients with intensive care unit experience. Unlike MIMIC-III, which stored all data in a collection of 26 data sheets, MIMIC-IV reflects the source of data by dividing the data into different modules, which are divided into six modules: Core, Hosp, ICU, ED, CXR, and Note. The module Core contains the basic information of all patients in the dataset, and the ICU module records the information collected from the clinical information system used in the ICU.

In this study, we aim to perform the in-hospital mortality prediction for the patient based on the patient's medical events produced during the first 48 hours of the ICU stays. Therefore, those patients who were hospitalized for less than 48 hours were excluded from our dataset, and for the MIMIC-IV dataset, experimental samples were only screened from patients with documented ICU admissions. To ensure the completeness of patient medical information, we remove the medical events with low frequency and maintain 27 vital sign measures, 70 prescription events, and 616 laboratory indexes. Finally, a final dataset containing 10,000 patients including 4306 positive patients who died in hospital is obtained. The screening procedures of the samples in the MIMIC-IV dataset is similar to that of the MIMIC-III dataset, and finally, 26 vital sign measures, 68 prescription events, and 66 laboratory indexes are retained. The brief description of our datasets is given in Table 2.

### 4.2. Baseline Methods

To evaluate the performance of TERTIAN, the following approaches are selected as baselines for comparison:

- TimeLine [19]: It is an attention-based interpretable deep learning model with time decaying for each visit, which uses an attention mechanism to aggregate context information of medical codes (diagnosis codes and procedure codes) and uses time-aware disease-specific progression function to model the influence of different historical visits on the patient's future health status for disease prediction
- GRUD [18]: It is a gated recurrent unit (GRU) based model for multivariate time series data modeling with missing values. In this model, two different trainable decays (input decay and hidden state decay) were set to capture the temporal patterns hidden in irregular time series
- IseeU [17]: It is a multiscale convolutional neural network for interpretable mortality prediction inside the ICU and uses the coalitional game theory to construct visual explanations to show how important these inputs are
- AttDMM [39]: It is a novel generative deep probabilistic model for predicting mortality risk in ICUs, which combines a deep Markov model with an attention mechanism to jointly capture long-term disease dynamics and different disease states in the health trajectory
- Transformer [40]: It is a mortality risk prediction model commonly composed of transformer. In this model, the encoder is mainly used to get the representation of the patient through the multihead attention mechanism
- GRASP [41]: It is a general framework for healthcare models, which defines similarities with different meanings between patients for different clinical tasks and finds similar patients with useful information accordingly. Then, it enhances the representation learning and prognosis of the given patient by leveraging knowledge extracted from similar patients.
- ConCare [20]: It is a health status representation learning framework for patients' clinical outcome prediction. In this model, a multichannel GRU with time-aware attention is used to adaptively learn the effect of time intervals between different medical records, and a multihead self-attention mechanism is deployed to capture the interdependencies among various medical information.

It should be noted that our study's aim is to use three types of historical clinical data (laboratory test results, vital signs, and prescription information) to predict the risk of in-hospital mortality in the future. However, the data used in the original baseline methods mentioned above are different. In order to facilitate a fair performance comparison, we modified the input part of these methods accordingly and uniformly adopted the zero-filling method to process the missing values. For TimeLine, GRUD, IseeU, and AttDMM, laboratory test results and vital signs are aggregated into time series data according to the actual sampling time, and prescription information is represented as a multihot vector and connected after the time series data. For GRASP, it can adopt any existing EHR representation learning model as the backbone model in its individual feature learning module. In our experiment, we implemented a version of GRASP that uses ConCare as the backbone for performance comparison. For ConCare, the input data of its original version is divided into sequential dynamic features (lab test values) and static baseline information (demographics and primary disease). In our experiment, we used laboratory test results and vital signs as sequential dynamic features and prescription information as static baseline information.

### 4.3. Metrics and Evaluation Strategy

We used precision, recall, *F*1-score, and Area under Curve (AUC) scores to evaluate the prediction performance. In general, AUC is a popular comprehensive score for binary classifier, and *F*1-score is the comprehensive evaluation index of precision and recall. We randomly selected 20% of the whole dataset as the test set and divided the rest into the training set and validation set in a ratio of 0.8 : 0.2. For each method, the experiments are repeated five times, and the average values with standard deviation for each evaluation metric are reported.

### 4.4. Implementation Details

We implemented all the methods based on the data extracted from MIMIC-III and MIMIC-IV with Keras 2.3.1 [42], the learning rate is set to 4*e* − 4, the RMSProp optimizer is used for training, the training batch size for MIMIC-III dataset is set to 64, and the training batch size is set to 128 for MIMIC-IV dataset. Training and testing are performed on a computer equipped with CPU: Intel (R) Xeon (R) Silver 4114, 128 GB RAM, GPU: Nvidia GeForce GTX 2080Ti 10 GB with CUDA 10.0. To avoid overfitting, early stop and dropout strategies are applied, and the dropout rate is set to 0.5. For the proposed TERTIAN, the dimensionality of attention query vectors and key vectors are set to 256, and the dimensionality of hidden state of GRU is set to 128, and the dropout rate for multihead attention is set to 0.2.

### 4.5. Results

#### 4.5.1. Performance of Mortality Prediction

Tables 3 and 4 present the performance of the different predictive approaches on the MIMIC-III dataset and MIMIC-IV dataset. According to the experimental results, we can see that the four methods (TERTIAN, ConCare, GRUD, and TimeLine) take into account the irregularity of time intervals in medical event sequences and achieve relatively good predictive performance. Among them, our method TERTIAN achieves *F*1-score of 0.9457 in the MIMIC-III dataset and 0.8666 in the MIMIC-IV dataset, which significantly outperforms the other baselines. On the one hand, it benefits from the introduction of time-aware transformer that can more accurately capture the unique temporal patterns of different clinical variable sequences. On the other hand, it is also due to the hierarchical attention mechanism used in TERTIAN, which fully explores the interaction and interrelationship among different types of data, thereby effectively improving the accuracy of the final patient fusion representation.

GRUD, TimeLine, and ConCare all assume that the impact weights of different medical events decrease with time and thus directly adopt the time decay-based approaches to model the patient's health progression patterns. Among them, the comprehensive performance of GRUD is higher than TimeLine, which may be because GRUD not only considers the time decay of input data but also the time decay of hidden state. For mortality prediction, however, the influence of different medical events on patient's health status does not completely follow such a monotonically decreasing pattern. Some medical indicators have a fluctuating relationship with the patient's health status. This affects the temporal feature representation capabilities of the above three methods. Moreover, it is easy to find that the performance of GRUD and TimeLine is relatively low in the methods which consider time irregularity. It is worth noting that the transformer learns the temporal characteristics of patient's historical medical data, which is more advantageous than other conventional temporal models. Therefore, the prediction performance of transformer is relatively good on both datasets. ConCare not only uses the time-aware attention weight function to capture the impacts of time intervals but also adopts a multihead self-attention mechanism with cross-head decoupling to effectively integrate the dynamic and static data while maintaining the diversity of features between heads. To a certain extent, this enhances the representation learning ability of the model for temporal sequences. As a result, ConCare obtains a precision of 0.9314 and an *F*1-score of 0.9275 in the MIMIC-III dataset and a precision of 0.8723 and an *F*1-score of 0.8572 in the MIMIC-IV dataset. GRASP, which takes ConCare as the backbone and not only uses ConCare to learn the feature representation of each patient but also leverages knowledge extracted from similar patients to enhance the representation learning of the patient, which improves the predictive performance, and the overall performance is second only to TERTIAN. Although AttDMM does not consider irregular time series, it jointly learns both long-term disease dynamics and different disease states in health trajectory, which is helpful to improve the performance of ICU prediction models. It is worth noting that although IseeU utilizes a multiscale convolutional neural network to learn local features under different time scales through multiple convolution kernels of different sizes, it cannot adequately capture the implicit fine-grained temporal features hidden in irregular time intervals. Therefore, its performance is the lowest of all comparison methods in both datasets.

#### 4.5.2. Benefits of Time-Aware Transformer Module

In this section, we first analyze the benefits of time-aware transformer used for temporal pattern learning on the predictive performance. We compare TERTIAN with its three variants. The first one is TERTIAN__GRU_, which is obtained by replacing the transformer in our model with GRU. The second is TERTIAN__temp_, which utilizes the positional encoding of transformer instead of time interval information. The third is TERTIAN__att_, which is obtained by removing the local-based attention mechanism immediately behind the transformer.

**Figure 2:**
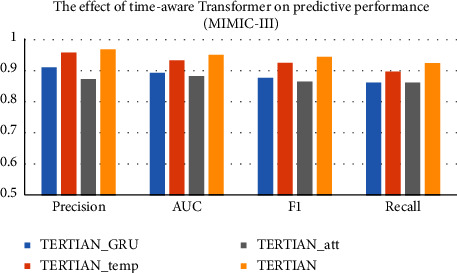
The effect of time-aware transformer on predictive performance (MIMIC-III).

**Figure 3:**
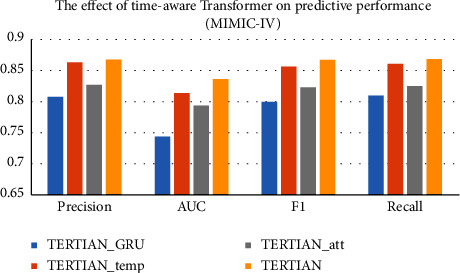
The effect of time-aware transformer on predictive performance (MIMIC-IV).

It can be seen from Figures 2 and 3 that the performance TERTIAN__temp_ is significantly lower than that of our model. It demonstrates that considering the irregularity of the time interval between medical events has an obvious promotion effect on modeling the patient's condition progression pattern. Moreover, when we replace the transformer in TERTIAN with GRU, the performance of the resulting variant model TERTIAN__GRU_ also shows a remarkable drop. Compared to TERTIAN in the MIMIC-III dataset, its precision, recall, AUC, and *F*1-score are decreased to 0.9109, 0.8625, 08951, and 0.8780, respectively, and in MIMIC-IV, its *F*1-score also decreased 6.69%. It indicates that the transformer has better capability for time series modeling than GRU. The main reason may be that the multihead attention mechanism inherent in the transformer can capture the relationships between medical events at any position in a sequence regardless of their distance, which makes it easier to learn long-term dependencies. Additionally, in our model, a local-based attention mechanism is deployed behind the transformer for future capturing of the influence weights of different historical medical records on the patient's future health status, so as to improve the accuracy of model's feature representation. From Figures 2 and 3, a decline in model's performance in both datasets can be clearly found when the local-based attention module behind the transformer is removed. In MIMIC-III, the performance of TERTIAN__att_ in terms of AUC and *F*1-score is significantly lower than that of TERTIAN by nearly 0.07, and in MIMIC-IV, the above two evaluation indicators both drop by about 0.04. This fully proves the effectiveness and necessity of the local-based attention mechanism.

#### 4.5.3. Effect of Hierarchical Feature Fusion

**Figure 4:**
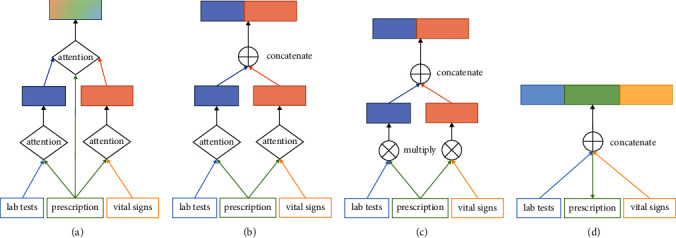
Different fusion methods. (a) TERTIAN. (b) TERTIAN__#_. (c) TERTIAN__*∗*_. (d) TERTIAN__&_.

In our model TERTIAN, we applied a hierarchical feature fusion approach based on the two-layer attention mechanism to mine the interrelationships among different types of clinical data and produced the final fusion representation. In order to investigate the effect of our proposed feature fusion method, we compare TERTIAN (as shown in Figure 4(a)) with other three variants that adopt various fusion modes. The first model is named TERTIAN__#_, which is obtained by modifying the attention mechanism in the second layer of TERTIAN's hierarchical fusion module to a concatenation operation, as shown in Figure 4(b). The second one is called TERTIAN__*∗*_, which is obtained by further changing the attention mechanisms in the first layer of TERTIAN__#_'s fusion module to elementwise multiplication, as shown in Figure 4(c). The third is TERTIAN__&_, as shown in Figure 4(d), and its fusion module is simplified to a direct concatenation of the representation vectors corresponding to various types of data.

**Figure 5:**
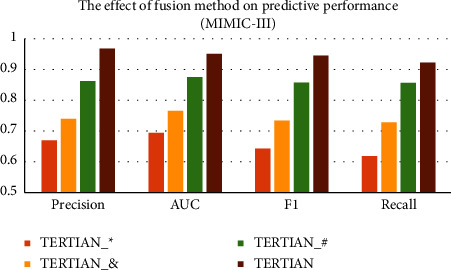
The effect of fusion method on predictive performance (MIMIC-III).

**Figure 6:**
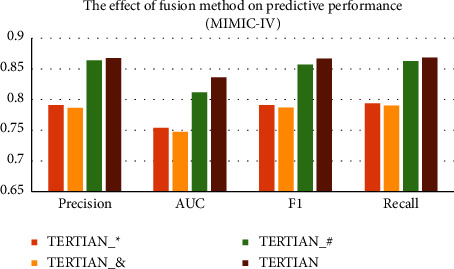
The effect of fusion method on predictive performance (MIMIC-IV).

From Figures 5 and 6, it can be clearly seen that the performance of TERTIAN__*∗*_ and TERTIAN__&_ are relatively poor (their evaluation results by all performance metrics are less than 0.8). This shows that the traditional fusion methods based on concatenation and/or element-wise multiplication cannot well capture the interdependence among different types of data and obtain effective feature fusion representation. In contrast, the introduction of the attention mechanism significantly improves the overall performance of the model. As shown in Figures 5 and 6, whether in the MIMIC-III dataset or MIMIC-IV dataset, the precision, recall, AUC, and *F*1-score of TERTIAN__#_ are all higher than those of TERTIAN__&_. In particular, the recall increased by 0.1258 in MIMIC-III and 0.0722 higher in MIMIC-IV. Our model TERTIAN adopts a double-layer attention mechanism to fully mine the complex interactions among various data, thereby effectively improving the accuracy of the final fusion representation. Therefore, its precision, recall, AUC, and *F*1-score are further increased to over 0.9 in the MIMIC-III dataset and around 0.85 in dataset MIMIC-IV, which are the highest among the four.

### 4.6. Parameter Sensitivity

In this section, we will further analyze the impact of several important parameters on the model performance. The first parameter is the dimension of key and query vectors in the two-layer attention mechanism, and the other is the dimension of hidden state in the GRU module used for prescription feature learning.

**Figure 7:**
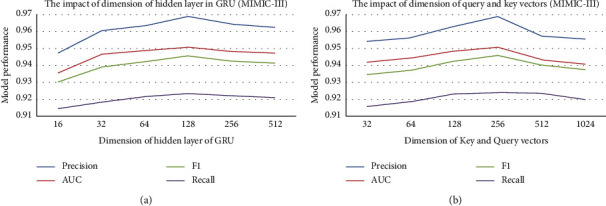
The impact of several important parameters on the model performance (MIMIC-III). (a) Sensitivity analysis for GRU. (b) Sensitivity analysis for attention.

**Figure 8:**
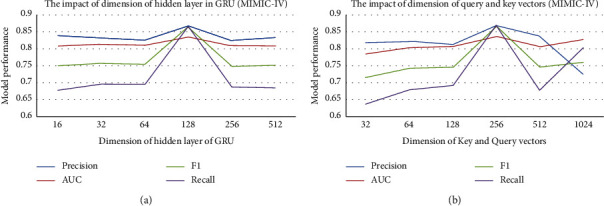
The impact of several important parameters on the model performance (MIMIC-IV). (a) Sensitivity analysis for GRU. (b) Sensitivity analysis for attention.

Figures 7 and 8 illustrate how the predictive performance of TERTIAN varies with the above-mentioned parameters on two datasets. It can be found that with the increase of each parameter value, the AUC of our model shows a similar trend of rising first and then falling. The main reason may be that when the vector dimension is too small, the feature information that the vector can express is very limited, which affects the accuracy of feature representation. With the increase of vector dimension, the feature representation ability of vector is enhanced, so the prediction performance of the model is also significantly improved. However, when the vector dimension continues to grow, the noise contained in the feature vector may also increase. Therefore, when the dimension exceeds a certain threshold, too much useless noise will reduce the effectiveness of the feature representation, which leads to the decline of model performance. In this paper, we finally determine the optimal values of these parameters according to the experimental results.

## 5. Conclusion

Risk prediction from EMRs is one of the key challenges in predictive health care. We have focused on the task of predicting ICU mortality events that take place more than 48 hours after admission. In this work, we proposed a novel deep learning model for clinical endpoint prediction. First, we introduce the time-aware transformer that automatically learns the irregular temporal pattern of medical events. Then, a hierarchical attention structure was proposed to capture the interaction between heterogeneous data and obtain a more comprehensive and accurate patient representation. Via performance comparisons with a suite of deep learning benchmarks, we demonstrated state-of-the-art results on real-world dataset (MIMIC-III and MIMIC-IV) and accounted for incremental sources of gains from various design choices. In addition, we further proved the effectiveness and advantages of each module of TERTIAN through two ablation experiments. In our future work, we will try to extend our model to other clinical risk prediction tasks to further verify its scalability and generalization capabilities and add interpretable modules to alleviate the limitations of the black-box model of deep learning.

## Figures and Tables

**Table 1:** Notations used in TERTIAN.

Symbol	Definition and description
*P*	Set of patients
*X* ^*l*^	Laboratory test results
*X* ^*r*^	Vital signs
*M*	Prescription information
*Z*	The total number of prescription codes
*C*	The category label about laboratory test results and vital signs
*d*_*it*_^*C*^	The timestamp of the *t*th observation *x*_*it*_^*C*^
*δ*_*it*_^*C*^	The interval between any two adjacent records.
*s*_*t*_	The normalized time interval vector
*f*_*t*_	The time-aware contextual feature representation of laboratory test results/vital signs
*g*_*t*_	The hidden representation of prescription information
*h*_*t*_^*l*^	The mixed representation of prescription information and laboratory test results
*h*_*t*_^*r*^	The mixed representation of prescription information and vital signs
Γ	The final patient fusion representation
*y*	The truth value
*y*′	The prediction label

**Table 2:** Statistics of the final dataset.

General profile	MIMIC-III	MIMIC-IV
#of patients (positive/negative)	10000 (4306/5694)	14344 (4631/9713)
#of unique lab events	616	66
#of unique vital sign indexes	27	26
#of unique prescription codes	70	68
Avg. # of unique lab events per patient	60.23	42.77
Avg. # of unique vital sign indexes per patient	11.06	20.92
Avg. # of unique prescription codes per patient	8.22	12.96

**Table 3:** Performance of mortality prediction task (MIMIC-III).

Model	Precision	AUC	*F*1	Recall
TimeLine	0.8510 (±0.0243)	0.8589 (±0.0288)	0.8384 (±0.0342)	0.8267 (±0.0449)
GRUD	0.8434 (±0.0071)	0.8835 (±0.0062)	0.8672 (±0.0069)	0.8925 (±0.0122)
IseeU	0.8164 (±0.0317)	0.8524 (±0.0061)	0.8323 (±0.0061)	0.8521 (±0.0331)
AttDMM	0.8554 (±0.0089)	0.8621 (±0.0161)	0.8582 (±0.0093)	0.8609 (±0.0098)
Transformer+	0.9387 (±0.0064)	0.9139 (±0.0096)	0.9044 (±0.0111)	0.8521 (±0.0173)
GRASP	0.9346 (±0.0089)	0.9495 (±0.0063)	0.9322 (±0.0091)	0.9243 (±0.0069)
ConCare	0.9314 (±0.0118)	0.9361 (±0.0074)	0.9275 (±0.0083)	0.9238 (±0.0131)
TERTIAN	0.9689 (±0.0075)	0.9506 (±0.0039)	0.9457 (±0.0043)	0.9236 (±0.0092)

**Table 4:** Performance of mortality prediction task (MIMIC-IV).

Model	Precision	AUC	*F*1	Recall
TimeLine	0.8332 (±0.0195)	0.8038 (±0.0265)	0.8332 (±0.0194)	0.8349 (±0.0180)
GRUD	0.8494 (±0.0183)	0.8288 (±0.0239)	0.8472 (±0.0173)	0.8469 (±0.0171)
IseeU	0.8316 (±0.0096)	0.8062 (±0.0261)	0.8211 (±0.0137)	0.8206 (±0.0130)
AttDMM	0.8252 (±0.0178)	0.8094 (±0.0117)	0.8242 (±0.0168)	0.8233 (±0.0192)
Transformer	0.8078 (±0.0031)	0.7783 (±0.0043)	0.8084 (±0.0029)	0.8091 (±0.0027)
GRASP	0.8747 (±0.0157)	0.8269 (±0.0194)	0.8622 (±0.0106)	0.8635 (±0.0155)
ConCare	0.8724 (±0.0082)	0.8260 (±0.0154)	0.8572 (±0.0119)	0.8677 (±0.0091)
TERTIAN	0.8680 (±0.0025)	0.8361 (±0.0093)	0.8666 (±0.0034)	0.8688 (±0.0028)

## Data Availability

The MIMIC-III data were used to support this study and are available at https://www.physionet.org/content/mimiciii/1.4/. The MIMIC-IV data were used to support this study and are available at https://www.physionet.org/content/mimiciv/1.0/.
